# Genetic Transformation of the Filamentous Fungus *Pseudogymnoascus verrucosus* of Antarctic Origin

**DOI:** 10.3389/fmicb.2019.02675

**Published:** 2019-11-22

**Authors:** Anaí Díaz, Pablo Villanueva, Vicente Oliva, Carlos Gil-Durán, Francisco Fierro, Renato Chávez, Inmaculada Vaca

**Affiliations:** ^1^Departamento de Química, Facultad de Ciencias, Universidad de Chile, Santiago, Chile; ^2^Departamento de Biología, Facultad de Química y Biología, Universidad de Santiago de Chile, Santiago, Chile; ^3^Departamento de Biotecnología, Universidad Autónoma Metropolitana, Unidad Iztapalapa, Mexico City, Mexico

**Keywords:** transformation, *Pseudogymnoascus*, protoplasts, electroporation, Antarctica

## Abstract

Cold-adapted fungi isolated from Antarctica, in particular those belonging to the genus *Pseudogymnoascus*, are producers of secondary metabolites with interesting bioactive properties as well as enzymes with potential biotechnological applications. However, at genetic level, the study of these fungi has been hindered by the lack of suitable genetic tools such as transformation systems. In fungi, the availability of transformation systems is a key to address the functional analysis of genes related with the production of a particular metabolite or enzyme. To the best of our knowledge, the transformation of *Pseudogymnoascus* strains of Antarctic origin has not been achieved yet. In this work, we describe for the first time the successful transformation of a *Pseudogymnoascus verrucosus* strain of Antarctic origin, using two methodologies: the polyethylene glycol (PEG)-mediated transformation, and the electroporation of germinated conidia. We achieved transformation efficiencies of 15.87 ± 5.16 transformants per μg of DNA and 2.67 ± 1.15 transformants per μg of DNA for PEG-mediated transformation and electroporation of germinated conidia, respectively. These results indicate that PEG-mediated transformation is a very efficient method for the transformation of this Antarctic fungus. The genetic transformation of *Pseudogymnoascus verrucosus* described in this work represents the first example of transformation of a filamentous fungus of Antarctic origin.

## Introduction

Antarctica, one of the most extreme ecosystems in the world, is inhabited by a myriad of microorganisms that have adapted to this harsh environment using several cellular and molecular strategies (Pearce, 2012). Among the microorganisms usually found in Antarctica, filamentous fungi are one of the most interesting groups. The number of fungal species recorded so far in the different Antarctic ecosystems studied is very high, suggesting a great diversity of fungi (Bridge and Spooner, 2012; Rosa et al., 2019). In addition, studies prospecting bioactivities in filamentous fungi isolated from Antarctica have gained much interest during last decades, showing that these fungi produce metabolites with interesting biological activities (Tian et al., 2017; Vaca and Chávez, 2019), as well as enzymes with novel properties, which could be useful for biotechnological purposes (Duarte et al., 2017, 2018).

Cold-adapted fungi from the genus *Pseudogymnoascus* are prevalent in most of the Antarctic environments (Ogaki et al., 2019; Rosa et al., 2019). To date, the species *P. appendiculatus*, *P. destructans*, *P. pannorum*, *P. verrucosus*, and *P. vinaceous* have been isolated from different Antarctic environments (Loque et al., 2010; Gomes et al., 2018; Alves et al., 2019; Kochkina et al., 2019; Rosa et al., 2019). Several studies have shown that extracts of these fungi produce bioactive metabolites with potential application, including antibacterial, antifungal, tripanocidal, herbicidal, and antitumoral activities (Furbino et al., 2014; Henríquez et al., 2014; Gonçalves et al., 2015; Gomes et al., 2018; Purić et al., 2018; Vieira et al., 2018). On the other hand, *Pseudogymnoascus* strains of Antarctic origin also have shown ability to produce cold-adapted enzymes with potential application in several industrial processes (Loperena et al., 2012; Poveda et al., 2018).

The aforementioned studies confirm the enormous biotechnological potential of *Pseudogymnoascus* strains of Antarctic origin. However, the future exploitation of this potential will require the development of suitable genetic tools. In this sense, and in order to study the genes responsible for the biosynthesis of secondary metabolites and enzymes from *Pseudogymnoascus* strains of Antarctic origin, an efficient transformation system is necessary. To the best of our knowledge, to date only an *Agrobacterium tumefaciens*-mediated transformation (ATMT) method has been used for the transformation of fungi from the genus *Pseudogymnoascus*. Zhang et al. (2015) used this method to perform the successful transformation of two different species: *Pseudogymnoascus destructans*, the etiologic agent of white-nose syndrome of hibernating bats, and *Pseudogymnoascus pannorum*, a species widely distributed in different environments.

ATMT has been described as a very laborious and time-consuming protocol that involves, among others, the construction of dual vectors for transformation and the previous transformation of bacteria *A. tumefaciens* (Rehman et al., 2016; Li et al., 2017). Thus, the development of alternative methods that could be simpler and faster is desirable and should be fostered (Rehman et al., 2016). Among alternative methods, polyethylene glycol (PEG)-mediated transformation has been highlighted as a simple and fast method, which has been successfully applied for the transformation of many fungal species (Rodríguez-Iglesias and Schmoll, 2015). On the other hand, electroporation has also been used for the transformation of several fungi. An additional advantage of this method is the possibility of using protoplasts, mycelia, or conidia, making electroporation potentially faster and simpler than protoplast transformation (Rivera et al., 2014).

Since several years, our team has been working in the study of the secondary metabolism and enzymes of Antarctic fungi, including species from the genus *Pseudogymnoascus*. As a result, we have described and purified novel secondary metabolites with interesting properties from these strains (Henríquez et al., 2014; Figueroa et al., 2015). In addition, we detected that these strains produce cold-adapted enzymes (Poveda et al., 2018). Unfortunately, at genetic level, the lack of a suitable transformation system hinders to go further in our research. To overcome this difficulty, we have addressed the development of transformation methods for *Pseudogymnoascus* strains of Antarctic origin. In this work, we have applied these developments, performing the successful transformation of an Antarctic strain, *Pseudogymnoascus verrucosus*, by using two different methodologies: PEG-mediated transformation and electroporation of germinated conidia. To the best of our knowledge, this is the first report of successful transformation in *P. verrucosus*, and more important, the first example of transformation of a filamentous fungus of Antarctic origin.

## Materials and Methods

### Fungal Strain, Culture Media, and Plasmid

The strain *P. verrucosus* FAE27 used in this work was previously isolated from an Antarctic marine sponge (Henríquez et al., 2014). The fungus was routinely kept on potato dextrose agar (PDA, Difco) and grown at 15°C. CM medium (6 g/l yeast extract, 6 g/l casein acid hydrolysate, 10 g/l sucrose) was used for growing the fungus in flasks.

The plasmid used for transformation experiments was pAN7-1, which contains the hygromycin B phosphotransferase (*hph*) gene as a dominant selectable marker under the control of *gpd*A promoter and *trp*C terminator from *Aspergillus nidulans* (Punt et al., 1987). The plasmid was purified from *Escherichia coli* using routine procedures and dialyzed against distilled water prior to use. In the case of experiments requiring linearized DNA, the plasmid was previously digested with *Hin*dIII.

### Antibiotic Resistance Test of *P. verrucosus* FAE27

To determine the sensitivity of *P. verrucosus* FAE27 toward hygromycin B and phleomycin, 1 × 10^7^ protoplasts (see section “Protoplast Isolation”) were added to 7.5 ml of molten overlay agar (CM medium plus 1% sorbitol and 0.8% agar) supplemented with different concentrations of hygromycin B (1, 2.5, 5, 10, and 20 μg/ml) or phleomycin (50, 100, 200, 500, and 700 μg/ml). Each mixture was quickly poured onto 7.5 ml of base agar (CM medium plus 1% sorbitol and 2%) on Petri dishes. Dishes were incubated during 15 days at 15°C. After this time, the presence of hygromycin-resistant or phleomycin-resistant colonies was evaluated. The same procedure was performed using 1 × 10^7^ germinated conidia instead of protoplasts.

### Polyethylene Glycol-Mediated Transformation of *P. verrucosus* FAE27

#### Protoplast Isolation

*P. verrucosus* FAE27 was cultured on PDA plates at 15°C for 15 days. Asexual spores (conidia) of one plate were harvested by gently scraping the agar with sterile distilled water. The resulting spore suspension was filtered through a sterile Miracloth filter (EMD Millipore, USA). The filtered spore suspension was inoculated in 50 ml of CM medium, and incubated at 15°C and 180 r.p.m. for 72 h. Mycelia obtained was collected by filtration in Miracloth filter and washed three times with 0.9% NaCl. Then, 1.5 g of harvested mycelia were aseptically transferred to an Erlenmeyer flask containing 10 ml of TPP solution (50 mM K_2_HPO_4_, 50 mM KH_2_PO_4_, 0.7 M KCl, pH 5.8). In parallel, 0.4 g of lysing enzymes from *Trichoderma harzianum* (Sigma-Aldrich, USA) were dissolved in 10 ml of TPP and filtered through 0.22 μm filter (Millex®GP, EMD Millipore, USA). This filtered solution was added to the Erlenmeyer flask containing resuspended mycelia, and the mixture was incubated at 28°C and 80 r.p.m. Protoplast release was checked every 30 min by counting in Neubauer chamber. Optionally, to increase the number of protoplasts released, 40 μl of β-glucuronidase solution (Sigma-Aldrich, USA) were added at 30 min, 1 h, and 2 h of incubation. Released protoplasts were carefully filtered two times, first using sterile Miracloth and then a sterile 40-μm nylon filter. The final solution containing protoplasts was centrifuged at 3,000 r.p.m. for 4 min, and the supernatant was discarded.

#### Polyethylene Glycol/CaCl_2_-Mediated Transformation

Protoplasts obtained were washed and centrifuged three times with KCM (0.5 M MES, 0.7 M KCl, 50 mM CaCl_2_, pH 5.8), and then resuspended with STC (20% sucrose, 10 mM Tris–HCl pH 8.0, 50 mM CaCl_2_) to 10^8^ protoplasts/ml. About 100 μl of protoplasts suspension (1 × 10^7^ protoplasts) were mixed with different amounts of plasmid DNA (3, 5, or 10 μg), 10 μl of 0.1 M aurintricarboxylic acid solution (Sigma-Aldrich, USA), and 10 μl of transformation solution PCM. Composition of PCM was 0.5 M MES, 50 mM CaCl_2_, and 50% PEG (PEG 3350, PEG 6000, or PEG 8000), pH 5.8.

Transformation mixtures were incubated on ice for 30 min, diluted with 500 μl of PCM, and incubated again at room temperature for additional 30 min. Then, 600 μl of STC and 3 ml of CM medium containing 1 M sorbitol (CM-sorbitol) were added. This mixture was incubated at 15°C and 80 r.p.m. during a protoplast recovery period of 24 h. After this time, 200–300 μl aliquots of transformation mixtures were added to 7.5 ml of molten overlay agar (CM medium, 1% sorbitol, 40 μg/ml hygromycin B, 0.8% agar). Mixture was quickly poured onto 7.5 ml of base agar (CM medium, 1% sorbitol, 40 μg/ml hygromycin B, 2% agar) on Petri dishes. After 15–20 days of incubation at 15°C, hygromycin-resistant colonies were visible. It is important to highlight that the temperature of molten agar must be around 40–42°C. Molten agar at higher temperatures dramatically reduces the number of transformants obtained, and at 45°C transformants are not obtained.

To verify viability of the protoplasts and effectiveness of the antibiotic selection during the experimental procedure, two controls were included: a transformation experiment without DNA and hygromycin B, and a transformation experiment without DNA but with hygromycin B.

#### Transformation Experiments Including Lithium Acetate

In some PEG-mediated transformations protocols previously published, calcium was replaced by other cations such as lithium (Mach, 2003; Olmedo-Monfil et al., 2004). Accordingly, we also performed PEG-mediated transformation experiments with solutions containing lithium acetate. In these experiments, we followed exactly the same protocol described for PEG/CaCl_2_-mediated transformation, changing one or all the solutions as follows:

1. Protoplasts obtained were washed and centrifuged three times with KLM (0.5 M MES, 0.7 M KCl, 50 mM lithium acetate, pH 5.8) and resuspended with STL (20% sucrose, 10 mM Tris-HCl pH 8.0, and 50 mM lithium acetate).
2. PCM was replaced by PLM. Composition of PLM was 0.5 M MES, 50 mM lithium acetate, and 50% PEG (PEG 3350, PEG 6000, or PEG 8000), pH 5.8.
3. After 30 min at room temperature, 600 μl of STL (not STC) were added.

### Electroporation of Germinated Conidia of *P. verrucosus* FAE27

#### Preparation of Germinated Conidia

*P. verrucosus* FAE27 was cultured on PDA plates at 15°C for 15 days. Asexual spores from four Petri dishes were harvested by scraping their surfaces with 5 ml of potassium phosphate buffer (0.03 M potassium phosphate, 0.02% Tween 20, pH 7.0). Mycelia fragments were removed by filtering through sterile Miracloth. The filtered conidia were concentrated by centrifugation at 9,000 r.p.m. for 15 min, washed three times with deionized water, and suspended in water to 10^8^ conidia/ml. Conidia suspension was kept at 4°C for 24 h to induce dormancy and thus obtain a synchronized germination. For germination, 50 ml of CM medium were inoculated with conidia at a final concentration of 1 × 10^7^ conidia/ml, and incubated at 15°C and 180 r.p.m. during 19–20 h. After this time, 90–95% of spores were germinated.

Germinated conidia were centrifuged at 6,000 r.p.m. during 15 min, resuspended in 1 ml of ice-cold distilled water, and collected again by centrifugation during 15 min at 8,000 r.p.m. and 10°C. Then, the germinated conidia were resuspended to 1 × 10^8^ conidia/ml in an ice-cold osmotic buffer: HEPES buffer (1 mM HEPES pH 7.5, 50 mM mannitol) or lithium acetate buffer (10 mM Tris-HCl pH 7.5, 270 mM sucrose, 1 mM lithium acetate).

#### Treatment of Germinated Conidia With Dithiothreitol

In some experiments, germinated conidia were treated with a DTT solution before their resuspension in osmotic buffer. DTT solution (50 mM potassium phosphate buffer, 25 mM DTT, pH 7) was prepared and sterilized by filtration. The conidia were resuspended in 1 ml of DTT solution and incubated at 15°C and 60 r.p.m. for 15 min to be subsequently centrifuged and resuspended in the osmotic buffer.

#### Electroporation of Germinated Conidia

Aliquots of 100 μl containing 1 × 10^7^ germinated conidia were mixed with 3, 5, or 10 μg of plasmid DNA and kept on ice for 15 min before electroporation. Each transformation mixture was transferred into a 0.2 cm electroporation cuvette and subjected to electroporation using the Gene Pulser Xcell Total System (Bio-Rad Laboratories, USA). Each transformation mixture was electroporated by a single exponential pulse ranging between 0.8 and 2.0 kV/cm, whereas capacitance and resistance were kept constant at 25 μF and infinite Ω resistance, respectively. Experiments using capacitances of 10 or 50 μF, or lower resistances, were also performed.

Immediately after pulse, 1 ml of ice-cold CM-sorbitol medium was added to each cuvette. Each mixture was transferred to a sterile tube. Then, 1 ml of CM-sorbitol was added again and each mixture was incubated at 15°C for 24 h and 80 r.p.m. for recovery. Finally, each transformation mixture electroporated was poured on Petri dishes exactly as was described for protoplast transformation (see section “Polyethylene Glycol/CaCl_2_-Mediated Transformation”). Hygromycin-resistant colonies were visible after 15–20 days of incubation at 15°C.

For all the transformation experiments, the same controls described in PEG/CaCl_2_-mediated transformation were performed. In addition, controls where the pulse was omitted were included.

### Stability Test for Hygromycin-Resistant Transformants

The mitotic stability of the transformants was assessed. For this purpose, 10 hygromycin-resistant transformants obtained by PEG-mediated transformation and 10 hygromycin-resistant transformants obtained by electroporation were randomly selected and transferred on plates with selective medium (CM medium containing 40 μg/ml hygromycin B). In order to obtain monosporic cultures of these transformants, asexual spores (conidia) were collected, diluted, and plated on selective media again. After growth, conidia from a single colony were transferred to non-selective medium (CM medium without hygromycin B) and incubated 2 weeks at 15°C. This last step was successively repeated three times. Finally, conidia growing on non-selective medium were transferred back to selective medium to confirm their resistance toward hygromycin B.

### Molecular Analysis of Hygromycin-Resistant Transformants

Genomic DNA from the hygromycin-resistant transformants was extracted using the protocol described by Navarrete et al. (2009). The presence of *hph* gene in DNA samples was detected by PCR using primers pgpdRNAi (GCTACATCCATACTCCATCC) and P4-sh2hindIII (AGTCGTAAGCTTAATGTGTGTCCTGTAGGCTT). PCR conditions were as follows: initial denaturation at 94°C for 3 min followed by 30 cycles at 94°C for 40 s, annealing at 55°C for 40 s and extension at 72°C for 2 min. The expected amplicon size was 1,689 bp.

## Results

### Sensitivity of *P. verrucosus* FAE27 Toward Different Antibiotics

Before transformation experiments, the sensitivity of *P. verrucosus* FAE27 toward hygromycin B and phleomycin was tested. We selected these antibiotics because they are widely used as selection markers in fungal transformation (Ruiz-Díez, 2002). Typically, phleomycin inhibits growth of filamentous fungi at concentrations of 25–150 μg/ml. In our case, we did not observe inhibition of *P. verrucosus* FAE27 growth using phleomycin, even at concentration as high as 700 μg/ml.

**Figure 1:**
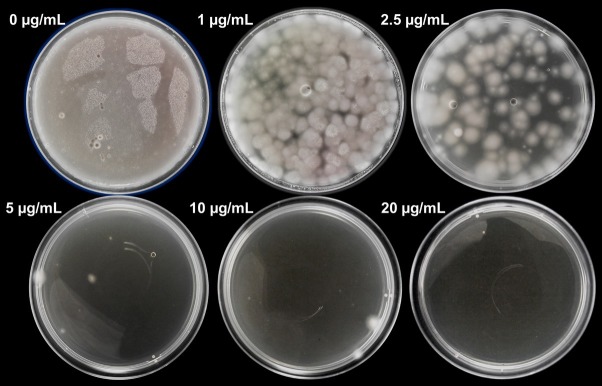
Sensitivity of protoplasts of *P. verrucosus* FAE27 toward hygromycin B. CM medium was supplemented with different concentrations of hygromycin B (0–20 μg/ml) and incubated for 15 days at 15°C. As can be seen, 20 μg/ml of hygromycin B completely inhibited fungal growth. The same experiment with identical results was performed using germinated conidia (data not shown).

In the case of hygromycin B, the growth of *P. verrucosus* FAE27 was inhibited at 20 μg/ml (Figure 1). This result agrees with those observed in *P. destructans* and *P. pannorum* (Zhang et al., 2015; see section “Discussion”). Therefore, hereafter we used 40 μg/ml of hygromycin B, equivalent to two fold the MIC determined, for the selection of transformants.

### Polyethylene Glycol-Mediated Transformation of *P. verrucosus* FAE27

#### Generation of Protoplasts

The first step in PEG-mediated transformation is the generation of protoplasts. For this purpose, fungal cell wall should be digested using hydrolytic enzymes. To the best of our knowledge, preparation of protoplasts has not been reported in any *Pseudogymnoascus* strain so far. In our case, we tested whether the use of lysing enzymes from *Trichoderma harzianum*, which are widely used to produce fungal protoplasts, provides good release of protoplasts from *P. verrucosus* FAE27. We observed that the incubation of mycelia with lysing enzymes yielded 3.82 × 10^8^ ± 0.86 × 10^8^ protoplasts after 3 h, enough to perform around 20 transformation experiments. This value was estimated considering 1 × 10^7^ protoplasts for each transformation experiment, amount optimal in most protocols of fungal PEG-mediated transformation (Liu and Friesen, 2012; Wang et al., 2018).

In addition to lysing enzymes, it has been described that the supplementation with β-glucuronidase improves protoplasts release from fungi (Wiebe et al., 1997). In *P. verrucosus* FAE27, we observed that the joint use of lysing enzymes and β-glucuronidase produced 9.26 × 10^8^ ± 1.36 × 10^8^ protoplasts, two to three times more protoplasts than lysing enzymes alone. Thus, and depending on protoplasts required by the user, the use of β-glucuronidase could be considered optional.

#### Effect of Plasmid DNA Concentration, Recovery Time of Protoplasts, Molecular Weight of Polyethylene Glycol, and Metal Ion in Polyethylene Glycol-Mediated Transformation

**Figure 2:**
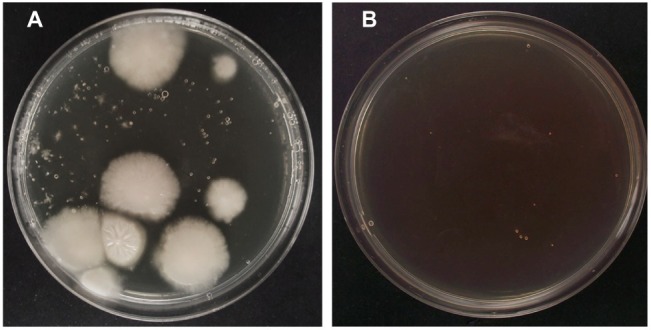
Successful transformation of *P. verrucosus* FAE27 by using PEG-mediated transformation method. **(A)** Transformation experiment using plasmid pAN7-1. Colonies observed after 20 days of growth on selective plates with 40 μg/ml hygromycin B. **(B)** Control transformation experiment without pAN7-1. Note the absence of fungal colonies.

For PEG-mediated transformation of fungi, researchers usually use DNA amounts ranging from 2 to 12 μg (Fincham, 1989; Wang et al., 2018). In our case, we tested three DNA amounts within this range (Table 1). As observed in Table 1, the increase of DNA amount from 3 to 5 μg increased the number of transformants obtained. However, in terms of transformation efficiency, this increase was not statistically significant. On the other hand, when DNA amount was increased up to 10 μg, the number of transformants increased around nine times, while transformation efficiencies increased around four times (Table 1). Therefore, for the rest of experiments, concentration of plasmid DNA was set at 10 μg. Figure 2 shows hygromycin-resistant transformants of *P. verrucosus* FAE27 obtained after a typical transformation experiment. Interestingly, the linearization of the plasmid negatively affected the efficiency of the transformation. Thus, and after several experiments using linearized plasmid, transformants were not obtained (Table 1).

**Table 1:** Effect of plasmid DNA amount on transformation efficiencies in PEG-mediated transformation of *Pseudogymnoascus verrucosus* FAE27.

Plasmid DNA (μg)	Number of transformants^a^	Transformation efficiency (transformants/μg DNA)^a^
3	8.67 ± 1.53	2.89 ± 0.51
5	15.67 ± 2.52	3.13 ± 0.50
10	134.0 ± 59.81	13.40 ± 5.98
10^b^	0	0

*In all experiments, PEG 3350 was used. Washing solution, resuspension solution and transformation solution contained 50 mM CaCl_2_*.

a *Standard errors were calculated from three independent experiments*.

b *DNA linearized with *Hin*dIII. This experiment was performed five times with identical results*.

Regarding time of regeneration of protoplasts after transformation, we used recovery times of 3 and 24 h. Transformants were obtained only after 24 h of recovery, so this time was used for all the experiments.

In PEG-mediated transformation of fungi, the components of the solutions used are important. Typical PEG-mediated transformation protocols include a washing solution which is used during the recovery process of the protoplasts, a solution for resuspension of protoplasts prior to transformation, and a transformation solution which is added together with DNA to perform the transformation. The main components of these solutions are an osmotic stabilizer to maintain the integrity of protoplasts during the procedure, calcium ions to produce the uptake of exogenous DNA by the cell, and PEG, which promotes cell agglomeration making DNA uptake easier. In particular, it has been observed that the molecular weight of the PEG is a key factor influencing the efficiency of transformation, so this parameter should be optimized in each fungal species (Rodríguez-Iglesias and Schmoll, 2015). Table 2 shows the effect of PEG 3350, PEG 6000, and PEG 8000 in the transformation efficiencies of *P. verrucosus* FAE27. As can be observed, best results were achieved by using PEG 3350. When molecular weight of PEG was increased, transformation efficiencies were drastically diminished and indeed, when PEG 8000 was used, transformants were not obtained (Table 2).

**Table 2:** Transformation efficiencies of PEG/CaCl_2_-mediated transformation of *Pseudogymnoascus verrucosus* FAE27, using PEG of different molecular weights.

Molecular weight of PEG within PCM solution	Number of transformants^a^	Transformation efficiency (transformants/μg DNA)^a^
PEG 3350	158.67 ± 51.62	15.87 ± 5.16
PEG 6000	37.00 ± 4.36	3.70 ± 0.44
PEG 8000	0	0

*For details of composition of PCM, see section “Materials and Methods.” In all experiments, 10 μg of plasmid DNA were used. Washing solution, resuspension solution and transformation solution contained 50 mM CaCl_2_*.

a *Standard errors were calculated from three independent experiments*.

As mentioned, typical PEG-mediated transformation protocols use calcium ions to facilitate DNA uptake. However, in some fungi, it has been described that good transformation yields may be obtained using solutions containing lithium ion and PEG (Mach, 2003; Olmedo-Monfil et al., 2004). Accordingly, we tested the effect of changing CaCl_2_ by lithium acetate in the last step of PEG/CaCl_2_-mediated transformation (Table 3). Our results indicate that this change is not favorable for the PEG-mediated transformation of *P. verrucosus* FAE27, producing a diminished transformation efficiency of 23% with respect to CaCl_2_. At this point it should be highlighted that the use of PEG 6000 or PEG 8000, together with lithium acetate, did not yield any transformant in several experiments performed (Table 3), indicating a joint detrimental effect of high molecular weight PEG and lithium acetate.

**Table 3:** Effect of changing PCM by PLM in the last step of PEG/CaCl_2_-mediated transformation of *Pseudogymnoascus verrucosus* FAE27.

Transformation buffer used	Number of transformants^a^	Transformation efficiency (transformants/μg DNA)^a^
PCM (containing 50 mM CaCl_2_)	158.67 ± 51.62	15.87 ± 5.16
PLM (containing 50 mM lithium acetate)	30.33 ± 5.86	3.03 ± 0.59

*In both experiments, PEG 3350 (optimal yield, see Table 2) and 10 μg of plasmid DNA (optimal yield, see Table 1) were used. PLM containing PEG 6000 or PEG 8000 were assayed in the same conditions but they did not yield transformants*.

a *Standard errors were calculated from three independent experiments*.

Similar negative results were observed when we performed experiments where CaCl_2_ was replaced by lithium acetate in all the solutions used, that is, a PEG/lithium acetate-mediated transformation. After several attempts, we never obtained transformants by using this method, even when PEG 3350 was used.

In conclusion, high efficiencies in PEG-mediated transformation of *P. verrucosus* FAE27 were successfully achieved using 10 μg of non-linearized DNA, and solutions containing low molecular weight PEG and CaCl_2_.

### Transformation of *P. verrucosus* FAE27 by Electroporation of Germinated Conidia

#### Optimization of Electroporation Conditions

Capacitance and field strength are critical parameters to obtain high transformation efficiencies in electroporation. Regarding capacitance, we initially tested three different capacitance values: 10, 25, and 50 μF. After several experiments, we observed that transformants were obtained only when 25 μF were used. On the contrary, experiments performed with capacitances of 10 or 50 μF did not give positive results. Therefore, the rest of experiments were done with capacitance value set at 25 μF.

In addition, we tested voltages in the range of 0.8–2.0 kV. Transformants were never obtained using high voltages of 1.5 and 2.0 kV (Table 4). Conversely, transformants of *P. verrucosus* FAE27 were obtained using voltages of 0.8, 1.0, and 1.25 kV (Table 4), corresponding to field strengths of 4, 5, and 6.25 kV/cm, respectively. We found that a voltage of 1.0 kV produced the highest electrotransformation efficiency, while pulses of 0.8 and 1.25 kV reduced the efficiency. In summary, the highest transformation efficiencies were obtained with 25 μF and 5 kV/cm.

**Table 4:** Effect of voltage and electroporation buffer on efficiencies of electroporation of germinated conidia of *Pseudogymnoascus verrucosus* FAE27.

Electroporation buffer	Voltage (kV)^a^	Number of transformants^b^	Transformation efficiency (transformants/μg DNA)^b^
Lithium acetate buffer	0.8	2.33 ± 1.53	0.78 ± 0.51
	1	4.00 ± 2.00	1.33 ± 0.67
	1.25	≤1.25	≤0.42
HEPES buffer	0.8	≤1.25	≤0.42
	1	≤0.91	≤0.30
	1.25	0	0

Capacitance at 25 μF, resistance ∞ Ω and 3 μg of plasmid DNA were used for all experiments. For details of composition of electroporation buffers, see section “Materials and Methods.”

a *Voltages of 1.5 and 2.0 kV were also used, but did not yield transformants*.

b *Standard errors were calculated from three independent experiments*.

On the other hand, the permeability of the cellular membrane depends on electroporation buffer composition (Dermol et al., 2016). Buffers commonly used in fungal transformation by electroporation are HEPES buffer (Chakraborty et al., 1991; Cruz-Ramón et al., 2019) and lithium acetate buffer (Xu et al., 2014; Vela-Corcía et al., 2015). We analyzed the effect of both buffers in the transformation efficiencies of *P. verrucosus* FAE27. Table 4 shows that higher transformation efficiencies were obtained using lithium acetate buffer in the three voltages assayed.

#### Effect of Amount of DNA and Treatment of Germinated Conidia With Dithiothreitol in Electrotransformation Efficiencies

In general, amount of DNA used in electroporation protocols ranges between 1 and 10 μg (Chakraborty et al., 1991; Dantas-Barbosa et al., 1998). We used three amounts of DNA within this range (Table 5). We observed that highest transformation efficiencies were achieved using 3 μg of plasmid (1.00 ± 0.33 transformants/μg DNA, Table 5). In addition, we observed that the linearization of the plasmid positively affected the efficiency of the transformation, which was increased to 2.67 ± 1.15 transformants/μg DNA (Table 5).

**Table 5:** Effect of plasmid DNA amount on efficiencies of electroporation of germinated conidia of *Pseudogymnoascus verrucosus* FAE27.

Plasmid DNA (μg)	Number of transformants^a^	Transformation efficiency (transformants/μg DNA)^a^
3^b^	8.00 ± 3.45	2.67 ± 1.15
3	3.00 ± 1.00	1.00 ± 0.33
5	1.67 ± 1.15	≤0.56
10	≤2	≤0.2

*Capacitance at 25 μF, resistance ∞ Ω and voltage of 1 kV were used for all experiments*.

a *Standard errors were calculated from three independent experiments*.

b *DNA linearized with HindIII*.

In previous reports (Meilhoc et al., 1990; Kawai et al., 2010; Cruz-Ramón et al., 2019), it has been described that DTT, a reagent that reduces disulfide bonds, promotes the weakening of fungal cell wall, thus facilitating DNA uptake in electroporation protocols. However, in our case, we observed that the treatment of germinated conidia with DTT had a negative effect on transformation efficiency, yielding 0.44 ± 0.19 transformants/μg DNA, around a 66% reduction in efficiency as compared to the control experiment without DTT.

### Mitotic Stability and Molecular Analysis of Transformants

The 20 randomly selected hygromycin-resistant transformants had high stability. After cultivation on non-selective medium for three successive times, they were able to grow well on selective plates containing hygromycin B. Importantly, these hygromycin-resistant transformants did not show any visible defect of growth as compared to wild-type *P. verrucosus* FAE27.

**Figure 3:**
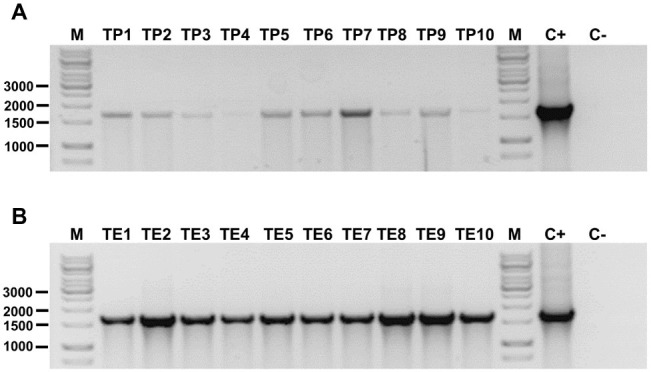
Detection of *hph* gene in transformants of *P. verrucosus* obtained by PEG-mediated transformation **(A)** or by electroporation of germinated conidia **(B)**. In each case, 10 transformants were analyzed. Lanes TP1 to TP10 and TE1 to TE10: Amplicon of 1,689 bp belonging to the *hph* gene conferring resistance to hygromycin B to the transformants. Lane C+: Positive control of amplification using pure pAN7-1. Lane C−: Negative control using genomic DNA from untransformed *P. verrucosus* FAE27. Lane M: Molecular-size marker (GeneRuler 1 kb DNA Ladder, Thermo Scientific). Relevant molecular weights are indicated.

Finally, the presence of *hph* gene in genomic DNA of the 20 randomly selected hygromycin-resistant transformants was verified by using PCR. Figure 3 shows all these DNA samples yielded the expected amplicon of 1,689 bp, whereas amplicon was not obtained using genomic DNA from untransformed *P. verrucosus*. These results confirm the successful genetic transformation of *P. verrucosus* FAE27.

## Discussion

In filamentous fungi, the most widely methods used for transformation are ATMT, PEG-mediated protoplast transformation, and electroporation (Li et al., 2017). To date, only ATMT has been used for the transformation of *Pseudogymnoascus* species (Zhang et al., 2015, 2018). On the contrary, and despite PEG-mediated transformation systems have been used to transform successfully a great variety of fungi (Rodríguez-Iglesias and Schmoll, 2015), to date this method has not been tested in *Pseudogymnoascus*. The PEG-mediated transformation method developed in this work for *P. verrucosus* FAE27 yielded an average of 159 transformants per transformation experiment (Table 2). As comparison, ATMT method yielded an average of 60 transformants in *P. destructans*, and 120 transformants in *P. pannorum* for each transformation experiment (Zhang et al., 2015, 2018). Thus, the number of transformants obtained with our PEG-mediated transformation method compares positively with those obtained by ATMT method, making our method an alternative that could be applied to other *Pseudogymnoascus* species.

Before transformation experiments, we evaluated the sensitivity of *P. verrucosus* FAE27 to hygromycin B and phleomycin. Our results showed that hygromycin B, at concentration of 20 μg/ml, completely inhibited the growth of *P. verrucosus* FAE27. Hygromycin B was previously used as selection marker for *P. destructans* and *P. pannorum*, which were inhibited at concentration of 100 μg/ml (Zhang et al., 2015). Thus, *P. verrucosus* FAE27 seems to be more sensitive to hygromycin B than *P. destructans* and *P. pannorum*. On the other hand, we observed that *P. verrucosus* FAE27 was able to grow in medium containing up to 700 μg/ml of phleomycin, which suggest that this fungus is essentially resistant to this antibiotic. These results suggest that for the future development of transformation systems in other *Pseudogymnoascus* species, hygromycin should be considered as the first option.

PEG-mediated transformation requires the generation of a suitable amount of protoplasts. In *P. verrucosus* FAE27, the digestion with lysing enzymes released in the order of 10^8^ protoplasts per gram and a half of mycelia, which is similar to the protoplasts obtained in other fungi successfully transformed (Liu and Friesen, 2012; Li et al., 2017). Interestingly, this number was dramatically increased by the addition of β-glucuronidase, a fact that has also been observed in other fungi (Wiebe et al., 1997).

Two essential components of solutions used in PEG-mediated transformation are PEG and metal cation. PEG induces disorder charges on the cytomembrane surface of protoplasts, thus altering membrane permeability and facilitating the entry exogenous nucleic acids into the cells (Fincham, 1989). In PEG-mediated transformations of fungi, in general it has been observed that low molecular weight PEG has superior performance than high molecular weight PEG, although this variable has to be optimized case-by-case (Becker and Lundblad, 2001). In our case, and in agreement with literature, we observed that in *P. verrucosus* FAE27, PEG of low molecular weight (PEG 3350) produced markedly better transformation efficiencies of protoplasts than high molecular weight PEG. Regarding metal ions, PEG-mediated transformation solutions usually contain calcium ions, which are thought opening channels in the cytomembrane, thereby facilitating the entry of exogenous DNA into protoplasts (Li et al., 2017). On the other hand, and although not much is known about the effect of lithium ions on membranes, it has been observed that these ions can alter membranes allowing DNA uptake into the cells (Jakobsson et al., 2017). In our experiments, we found that the replacement of calcium ions by lithium ions in the transformation solution produced a drastic reduction in transformation efficiencies. Indeed, the replacement of calcium by lithium in all the transformation solutions did not yield transformants. During the course of these experiments with lithium, we observed that control dishes of protoplasts viability showed around 1% of protoplasts regeneration, a very low level of regeneration as compared with experiments performed with calcium ions, where around 40% of protoplasts regenerated. Although more research is needed, these results suggest that the detrimental effect of lithium ions could be related with the lack of stability of the protoplasts in solutions containing lithium, or their inability to perform cell wall regeneration after transformation process.

We also identified other two key parameters that critically influenced the transformation efficiencies of *P. verrucosus* FAE27: temperature of molten agar, and time of recovery of transformants. Regarding the temperature of molten agar, most fungal transformation protocols described in literature specify that transformed protoplasts or electroporated conidia should be mixed with molten agar at 50°C (Gangavaram et al., 2009; Liu and Friesen, 2012; Cruz-Ramón et al., 2019). However, in our case, we never obtained transformants of *P. verrucosus* FAE27 using molten agar at temperatures over 45°C. On the contrary, good transformation efficiencies were always obtained with molten agar not exceeding 42°C. Important, and although our protocol of electroporation of germinated conidia had lower efficiencies (Tables 4, 5), the precautions regarding the temperature of molten agar also apply to this protocol. Taken together, these results suggest that both protoplasts and germinated conidia of *P. verrucosus* FAE27 are very sensitive to high temperatures. *P. verrucosus* FAE27 has a temperature range of growth between 4 and 25°C with optimal growth at 15°C (Henríquez et al., 2014; Poveda et al., 2018), which agrees with this observation. However, it should be highlighted that some mesophilic fungi also requires molten agar at low temperature to successfully obtain transformants (Cho et al., 2013; Roth and Chilvers, 2019), so this is not a specific requirement of cold-adapted fungi.

After transformation, transformants need a time of incubation for the regeneration of the cell wall before plating on selection medium (Liu and Friesen, 2012). In most protocols involving mesophilic strains, this recovery time ranges between 2 and 5 h (Liu and Friesen, 2012; Xu et al., 2014). Accordingly, we initially attempted recovery times of 3 h, but we did not obtain transformants. We reasoned that due to its cold-adapted character, *P. verrucosus* FAE27 probably has slower growth velocity than mesophilic fungi and needs longer recovery times. Indeed, the growth velocity of *P. verrucosus* is 2.4 mm/day at 15°C, which is markedly lower than the growth velocity of several mesophilic fungal species, which are in a range of 4–10 mm/day (Sautour et al., 2002; Segers et al., 2016). Consequently, we attempted a longer time of recovery of 24 h with successful results. This longer recovery time was applied to PEG-mediated transformation and the electroporation of germinated conidia methods.

In some PEG-mediated transformation protocols, the use of linear plasmids is recommended to increase the frequency of transformation. However, results obtained are variable and depend on the fungal species used (Poyedinok and Blume, 2018). In our case, we observed that the linearization of plasmid, in combination with PEG-mediated transformation, did not yield transformants (Table 1). Other authors have observed significant decrease in transformation efficiencies when a linear vector is used (Herrera-Estrella et al., 1990). It has been argued that during PEG-mediated transformation, linear vectors would be more susceptible to degradation by exonucleases (Lopes et al., 2008), which would negatively affect the transformation efficiencies.

Finally, although we obtained transformants of *P. verrucosus* by electroporation of germinated conidia, this method had comparatively, a lower efficiency. Our results show that the electroporation protocol yielded an average of four transformants per experiment. Low frequencies of transformation by electroporation have been previously observed in other fungal genera (Rivera et al., 2014). These low frequencies of transformation can be attributed to different causes. One of these may be a low viability of conidia after being submitted to electrical pulses. In our experiments, we observed that after a pulse of 1.0 kV, the viability of the germinated conidia was around 38%. Other researchers have obtained high transformation efficiencies with similar levels of conidia viability (Sánchez and Aguirre, 1996; Cruz-Ramón et al., 2019), so this would not be a key factor determining the low efficiencies observed during electrotransformation of *P. verrucosus*. In addition, we observed that an increase in the amount of DNA used also did not increase transformation efficiencies (Table 5). Although the mechanism of electroporation of germinated conidia still remains unclear (Li et al., 2017), it has been suggested that cell wall of filamentous fungi acts as a natural barrier to uptake of DNA during electroporation (Chakraborty et al., 1991). Accordingly, we thought that probably, the cell wall of germinated conidia of *P. verrucosus* is a natural barrier to the uptake of DNA that the electroporation conditions used in this work cannot completely overcome. Anyway, and despite the lower efficiency of the electrotransformation method, its easiness and speed make it a good option for functional analyses where few transformants are required, such as gene overexpression.

In summary, this study describes for the first time the successful transformation of a filamentous fungus of Antarctic origin, *P. verrucosus*. The transformation protocols here developed will enable the genetic analysis of *Pseudogymnoascus* strains of Antarctic origin.

## Data Availability Statement

All datasets generated for this study are included in the article/supplementary material.

## Author Contributions

IV, RC, and FF designed the experiment. AD, PV, VO and CG-D performed all laboratory analysis and evaluated the data. IV and RC drafted the manuscript.

### Conflict of Interest

The authors declare that the research was conducted in the absence of any commercial or financial relationships that could be construed as a potential conflict of interest.
